# MRI as a screening tool for prostate cancer: current evidence and future challenges

**DOI:** 10.1007/s00345-022-03947-y

**Published:** 2022-02-28

**Authors:** Christoph Würnschimmel, Thenappan Chandrasekar, Luisa Hahn, Tarik Esen, Shahrokh F. Shariat, Derya Tilki

**Affiliations:** 1 Martini-Klinik Prostate Cancer Center, University Hospital Hamburg-Eppendorf, Martinistraße 52, 20246 Hamburg, Germany; 2 Department of Urology, Lucerne Cantonal Hospital, Lucerne, Switzerland; 3 Department of Urology, Thomas Jefferson University, Philadelphia, USA; 4 Department of Urology, Koc University Hospital, Istanbul, Turkey; 5 Department of Urology, Comprehensive Cancer Center, Medical University of Vienna, Vienna, Austria; 6 Department of Urology, Weill Cornell Medical College, New York, NY USA; 7 Department of Urology, University of Texas Southwestern, Dallas, TX USA; 8 Department of Urology, Second Faculty of Medicine, Charles University, Prague, Czech Republic; 9 Institute for Urology and Reproductive Health, Sechenov University, Moscow, Russia; 10 Department of Urology, University Hospital Hamburg-Eppendorf, Hamburg, Germany

**Keywords:** Magnetic resonance imaging, MRI, Prostate cancer screening, Prostagram

## Abstract

**Purpose:**

Prostate cancer (PCa) screening, which relies on prostate-specific antigen (PSA) testing, is a contentious topic that received negative attention due to the low sensitivity and specificity of PSA to detect clinically significant PCa. In this context, due to the higher sensitivity and specificity of magnetic resonance imaging (MRI), several trials investigate the feasibility of “MRI-only” screening approaches, and question if PSA testing may be replaced within prostate cancer screening programs.

**Methods:**

This narrative review discusses the current literature and the outlook on the potential of MRI-based PCa screening.

**Results:**

Several prospective randomized population-based trials are ongoing. Preliminary study results appear to favor the “MRI-only” approach. However, MRI-based PCa screening programs face a variety of obstacles that have yet to be fully addressed. These include the increased cost of MRI, lack of broad availability, differences in MRI acquisition and interpretation protocols, and lack of long-term impact on cancer-specific mortality. Partly, these issues are being addressed by shorter and simpler MRI approaches (5–20 min bi-parametric MRI), novel quality indicators (PI-QUAL) and the implementation of radiomics (deep learning, machine learning).

**Conclusion:**

Although promising preliminary results were reported, MRI-based PCa screening still lack long-term data on crucial endpoints such as the impact of MRI screening on mortality. Furthermore, the issues of availability, cost-effectiveness, and differences in MRI acquisition and interpretation still need to be addressed.

##  Introduction

Prostate cancer (PCa) detection has historically relied primarily on prostate-specific antigen (PSA) testing [1]. Suspicious PSA values and/or suspicious digital rectal examination would trigger prostate biopsy (PBx). However, the “PSA pathway” is criticized for its weak sensitivity and specificity for clinically significant PCa diagnosis, which may result in over-diagnosis, and therefore overtreatment, of clinically insignificant PCa [2, 3]. For this reason, the United States Preventive Services Task Force recommended against PCa screening relying on PSA in 2012; this was partly retracted in 2018, based on the established potential benefits of PSA screening with regard to lower PCa-specific mortality [4, 5]. However, the true cancer-specific survival benefit of relying on PSA testing/screening is ambiguous and should be weighed against negative quality of life impact of PCa treatments. PSA screening should only be performed in well-informed men, taking into account patients’ expectations, comorbidities and life expectancy [6]. Magnetic resonance imaging (MRI) of the prostate has evolved to be an integral part of the initial radiographic evaluation for patients at risk for PCa, together with PSA testing and digital rectal examination [7]. By identifying suspicious regions within the prostate and targeting these lesions on MRI-fusion guided PBx, detection rates of clinically significant PCa are increased, while detection rates of clinically insignificant PCa are reduced compared to a standard trans-rectal or trans-perineal ultrasound-guided PBx [8]. For this reason, recent European Association of Urology (EAU) guidelines recommend performing MRI prior to any PBx in men at elevated risk for PCa [6]. Therefore, the combination of PSA testing, digital rectal examination and prostate MRI represents current best practice in the evaluation of thoroughly informed men at risk for PCa. Furthermore, the EAU guidelines also incorporated established individual risk factors which should be taken into account when considering PCa diagnostics, such as positive family history, race/ethnicity and/or germline mutations such as BRCA2 [6]. With the additional diagnostic power of MRI, the over-diagnosis of clinically insignificant PCa and, subsequently, overtreatment could be reduced [8, 9].

While the combined “MRI-PSA-pathway” standard is now increasingly utilized, recent pilot studies have begun to question whether PSA testing can be replaced by MRI altogether for the purpose of PCa screening [10, 11]. It is hypothesized that this “MRI-only-pathway” reduces the need for invasive PBx without sacrificing the ability to detect clinically significant PCa (Table 1). Furthermore, it might be conceivable that the “MRI-only-pathway” may evolve to be the preferred method for PCa screening in future; this would resemble radiographic screening programs for other malignancies, such as the use of mammography for early detection of breast cancer. Currently, several trials evaluating the “MRI-only-pathway” are underway [12–14]*.* This narrative review aims to provide a contemporary overview on the current evidence while discussing potential obstacles encountered when relying on prostate MRI as a screening tool for PCa. The search strategy for this non-systematic literature review included combinations of keywords covering the general topic of PCa screening and included both PSA-based and MRI-based approaches. PubMed, Scopus, Web of Science, Google Scholar and abstract pages of the latest EAU and American Urological Association congresses as well as clinical trial registration websites (clinicaltrials.gov, isrctn.com) were used for literature retrieval. Reference lists of appropriate publications were screened for potential further relevant publications. Finally, relevant publications were discussed among authors for eligibility of inclusion within this review.

**Table 1:** Prevalence of clinically significant prostate cancer (csPCa) according to prostate-specific antigen (PSA) values, derived from a secondary analysis of the Prostate, Lung, Colorectal, and Ovarian (PLCO) Cancer Screening Trial [40], as well as prevalence of csPCA according to PI-RADS scores 3–5, derived from the PRECISION trial [8] as well as the validation trial for the PI-RADS 2.0 system [41]

PSA value	csPCa prevalence
≤ 0.49–1.99 ng/ml	0.3–4.0%
2.0–3.99 ng/ml	8.5–12.4%
≥ 4.0 ng/ml	23.1%

PI-RADS score	csPCa prevalence
3	12.0–23.0%
4	49.0–60.0%
5	77.0–83.0%

csPCa was defined as any Gleason score ≥ 7, death from PCa, or T2b status after radical prostatectomy in the PLCO trial and as any Gleason score ≥ 7 in the PRECISION trial and the validation trial for PI-RADS 2.0

## Prostate cancer diagnosis with magnetic resonance imaging: technical considerations, image interpretation and pitfalls

When performing an MRI for PCa diagnosis, bi-parametric (bpMRI) or multi-parametric MRI (mpMRI) is employed. For both bpMRI and mpMRI, T2-weighted images are critical for precise imaging of prostate anatomy. Diffusion-weighted imaging (DWI), which assesses cell density, further helps distinguish cancerous and non-cancerous regions, since a higher cell density is encountered in cancerous regions [15]. In the third step, which is excluded in bpMRI, dynamic contrast enhancement (DCE), which relies on contrast agents, more clearly delineates the vascular supply within the prostate, which is preferentially taken up by malignant PCa [16].

The most employed reporting standard for prostate MRI follows the European Society of Urogenital Radiology guidelines. These guidelines (Prostate Imaging Reporting & Data System, PI-RADS), in their most recently updated and recommended PI-RADS version 2.1, assign a score to all concerning regions between grades one to five [17]. Based on this numerical grading, PI-RADS 1–2 are regarded as non-suspicious categories, PI-RADS 3 is regarded as “equivocal”, and PI-RADS 4–5 are regarded as suspicious [18]. PI-RADS 4–5 lesions are often “biopsy triggers” in clinical practice, since they predict clinically significant PCa with a high degree of certainty [18, 19]. On the other hand, PI-RADS 3 lesions have been associated with a variable rate of concordance with clinically significant PCa. In addition, the prevalence of PI-RADS 3 was reported between 6 and 39%, and some authors agree that the rate of PI-RADS 3 assignment by radiologists may serve as a surrogate parameter for understanding the MRI experience of the reader [20]. Excluding PI-RADS 1–2 on targeting can contribute to the reduction of unnecessary PBx [8, 18].

MRI for detection of PCa has been extensively evaluated and MRI-based fusion biopsy significantly improves detection rates of clinically significant PCa, compared to systematic biopsy alone [21–23]. For example, a recent randomized non-inferiority trial investigated the role of MRI-guided biopsies. Within this trial, Eklund et al. compared patients with a minimum PSA value of 3 ng/ml with one arm receiving a standard systematic biopsy and the other arm receiving a systematic biopsy and targeted biopsies of suspicious MRI findings. Eklund et al. reported a significantly higher rate of clinically significant PCa with MRI targeted biopsy, confirming the non-inferiority of the MRI-guided approach, despite lower rates of triggered biopsies in the experimental arm. [22]. Despite these important findings, the role of population-based PCa screening relying on MRI alone is underdeveloped and, therefore, not established. Some of the most important obstacles that still need to be addressed are the higher costs of mpMRI, limited availability and (time-consuming) expenditures of mpMRI, which is contradictory to the common principles of screening programs, which should be cost-effective, broadly available and most importantly, easy to apply and interpret [24–26]. To address cost-effectiveness, given the findings by Eklund et al., the combined benefit of detecting more clinically significant PCa while reducing rates of unnecessary biopsies using the MRI-based approach, the trade-off toward a potentially lower rate of overtreatment could hypothetically correspond to cost-savings [22]. Furthermore, some authors also advocate a simpler, faster and more cost-effective approach for prostate MRI, relying on bpMRI alone, which forgoes DCE and, therefore intravenous application of contrast agents, enabling faster acquisition times[27]. For example, in their meta-analysis of ten studies (*n* = 1705 patients) comparing bpMRI with mpMRI, Kang et al. could not detect statistically significant differences in PCa detection rates[28]. Furthermore, novel protocols with shorter acquisition times were also developed (“IMPROD”) and further validated in a prospective multi-institutional setting [29, 30]. Using a 15-min bpMRI protocol, Jambor et al. reported of a sensitivity of 97% (Confidence Interval [CI] 93–99%) and a negative predictive value of 95% (CI 87–98%) for the detection of Gleason Score ≥ 3 + 4 in systematic ± targeted biopsy. In another study by Weiss et al., acquisition times were even further lowered, down to 5 min [31]. Although this protocol was only tested on 52 patients within a single institution, the diagnostic accuracy was still reported to be comparable to standard mpMRI, which lays the foundation for a more feasible implementation of MRI within population-based PCa screening. Altogether, the potential of MRI as a potential screening tool still needs to be established, also in light of the higher demands and heterogeneity in interpreting MRI findings, that are far more challenging compared to relying on a simple PSA test alone.

## Future perspectives of magnetic resonance imaging for prostate cancer screening

Currently, several ongoing large-scale population-based trials are evaluating MRI for PCa screening (Table 2). A Canadian trial (NCT02799303), entitled MVP (MRI vs. PSA) Trial, randomized 525 men into MRI-only versus PSA for PCa screening, for which results are pending publication. To-date, only preliminary details on recruitment were reported and are available as a conference abstract presented at the 2021 EAU meeting [32]. A Swedish trial (ISRCTN54449243), entitled “GÖTEBORG-2”, which aims to recruit over 40,000 men, also investigates the benefit of MRI screening. However, this trial, with an estimated end date in 2040, relies on a combination of PSA and MRI screening [12], which therefore does not provide the strictest study endpoint, but might be more applicable in a broader population. In this regard, the ReIMAGINE trial, an MRI screening trial from a single institution in the United Kingdom (NCT04063566*),* uses an interesting primary outcome measure that includes acceptance rates of men for undergoing a 20-min MRI for screening purposes. Specifically, to assess this endpoint, men are randomly invited to take part in this trial at the level of consultations with general practitioners. Recently, ReIMAGINE reported an update stating that the recruitment goal had been reached (*n* = 309) and 303 men received a screening bpMRI and further results are being awaited [13]. All these above major trials will provide valuable information into the true prevalence of MRI lesions in a screening population and provide more insights with regard to the feasibility of MRI for population-based screening.

**Table 2:** Summary of ongoing trials for the overall comparison between prostate-specific antigen testing and magnetic resonance imaging for detection of prostate cancer within a screening routine

Author	Trial (Registration)	Location	Single/Multicentric	Study design (Arms)	Years of accrual	Accrual target, n	Inclusion criteria	Endpoints	Available results
Nam et al	Pilot study for MVP (NCT02799303)	Toronto (Canada)	Single center	1. mpMRI 2. PSA	06/2016–06/2020	47	Unselected men (general population) 50–75 years no family PCa history no prior Bx	Presence of PCa in Bx	mpMRI (AUC 0.81, 95% CI 0.67–0.94) as a stronger predictor for the diagnosis of PCa than PSA (AUC 0.67, 95% CI 0.52–0.84)
Nam et al	MVP (NCT02799303)	Toronto (Canada)	Single center	1. mpMRI 2. PSA	06/2016–06/2020	525	Unselected men (general population): $\ge$ 50 years No prior PSA in 3 years Normal DRE No prior Bx in 3 years No fam. history Asymptomatic	Primary: Clinically significant PCa Secondary: Clinically insignificant PCa	**Study under review** First updates at EAU 2021: approx. 50% less Bx Higher sign. PCa diagnosis
Kohestani et al	GÖTEBORG 2 (ISRCTN94604465)	Gotheburg (Sweden)	Single center	1. PSA $\ge$ 3 ng/ml bp/mpMRI + SB + TB 2. PSA $\ge$ 3 ng/ml bp/mpMRI + TB 3. PSA $\ge$ 1,8 ng/ml bp/mpMRI + TB	09/2015–12/2040	38,770	Men aged 50–60 from West Sweden identified from the Total Population Register	Primary: Reduction PCa mortality at 12 year Secondary: Risk reduction to detect insign. PCa PCa detection rates between bp and mpMRI	N/A
Eldred-Evans et al	IP1-PROSTAGRAM (NCT03702439)	London (UK)	Multicentric	1. PSA $\ge$ 3 ng/ml 2. 5-min non-contrast MRI 3. Ultrasonography + SB/ TB (pos. MRI)	10/2018 – 05/2019	408	Randomly selected men aged 50–69 years through 7 primary care practices in the UK	Primary: proportion of screening-pos MRI (PI-RADS 4–5) or ultrasonography or PSA test Secondary: number of clinically sig.and clinically insign. cancers detected (each test exclusively)	Detection of sign. PCa 2 × increased with MRI Trial was underpowered Statistically no significant differences (p = 0.71)
Auvinen et al	ProScreen (NCT03423303)	Helsinki (Finland)	Single center	Three levels of risk assessment • PSA • Kallikrein panel • MRI before the diagnostic procedure (prostate biopsy)	04/2021 - 12/2028	11,056	Randomly selected men aged 50–63 in Helsinki and Tampere	PCa mortality at 10 and 15 years of follow-up	N/A

*mp/bpMRI* multi-parametric/bi-parametric magnetic resonance imaging, *SB* systemic biopsy, *TB* targeted biopsy, *PCa* prostate cancer, *Bx* biopsy, *AUC* Area under the curve

Apart from these ongoing trials, some data are already available. The IP1-PROSTAGRAM trial, a cohort study including data from 408 patients from seven primary care practices and two imaging centers in the United Kingdom (NCT03702439), recently reported favorable performance characteristics for bpMRI as a community-based screening test [14]*.* Specifically, the blinded IP1-PROSTAGRAM study defined cut-offs for a positive MRI (PIRADS 3–5) versus cut-offs for a positive PSA test (≥ 3 ng/ml), either of which triggered a systematic PBx and additional fusion biopsy in the presence of suspicious lesions. In the comparison of positive PSA alone vs. positive MRI alone screening protocols, the latter was associated with higher rates of clinically significant PCa, with no associated increase in the rate of clinically insignificant PCa. These early findings strengthen the role of MRI for PCa screening compared to PSA alone. However, cautious interpretation of these early findings is necessary. First, no further large-scale population-based data are available. Second, MRI screening has yet to demonstrate that higher rates of detecting clinically significant PCa results in a measurable and meaningful impact on PCa-specific mortality compared to PSA screening programs or no screening at all. Therefore, the outcomes of the previously mentioned randomized trials, and further long-term observations of IP1-PROSTAGRAM, need to be awaited. Finally, the cost-effectiveness, availability and feasibility of MRI-based population screening remain issues that need to be resolved by health officials in future. Even if the issue of cost-effectiveness and availability are addressed, a major challenge for MRI screening remains providing a sufficient number of trained specialists for MRI acquisition and interpretation. Indeed, MRI acquisition and interpretation, despite the standardized PI-RADS v2.1 initiative, has a high rate of inter-observer variability and differences in common practice, especially in low-volume centers [33, 34]. This appears especially relevant when considering the “MRI-only-pathway”, since a misinterpretation of MRI findings due to lack of experience or incorrect MRI acquisition may potentially lead to under-detection and consequently under-treatment of clinically significant PCa. For this reason, from the current perspective, future pathways will most likely rely on the combination of both MRI and PSA pathways. Nevertheless, it is conceivable that the importance of MRI findings will increase compared to PSA testing within screening programs. Therefore, apart from the efforts to develop the necessary expertise to handle the growing demand of MRI acquisition and interpretation, novel and powerful artificial intelligence tools such as “machine learning/deep learning/radiomics” might be a feasible supplementary approach for tackling this demand in future [35–37]. Indeed, comparative studies already provide first experiences with deep learning that provided similar results to conventional clinical MRI interpretation [38]. In addition, the different standards in MRI acquisition need to be addressed in future. One major step in this direction was recently done by the PRECISION study group that proposed a new quality scoring system for MRI acquisition (PI-QUAL) [39]. The authors defined PI-QUAL as a score on a Likert scale from 1 to 5. PI-QUAL 1 implies no sequences are of any diagnostic quality, while 5 implies that each sequence is independently of optimal diagnostic quality. Therefore, the combination of this new quality assessment tool, eventually combined with artificial intelligence systems and further funding and distribution of well-trained MRI personnel and advanced MRI technology, the possibility of MRI-based PCa screening may be technically feasible.

## Conclusion

The implementation of MRI for prostate cancer diagnosis increased the diagnostic accuracy and helped reduce over-detection and overtreatment in clinical practice. Therefore, several trials are investigating the feasibility of “MRI-only” screening approaches, and assess if PSA testing could eventually be replaced within prostate cancer screening programs. Preliminary studies outline results that are in favor of the “MRI-only” approach. However, besides lack of long-term outcomes on crucial endpoints (such as the impact on cancer-specific mortality), further obstacles for implementing MRI-based screening programs (availability, cost-effectiveness, differences in MRI acquisition and interpretation) still need to be addressed.

## Data Availability

Not applicable.
